# Improvement in the traditional processing method and nutritional quality of traditional extruded cassava-based snack (modified Ajogun)

**DOI:** 10.1002/fsn3.43

**Published:** 2013-06-20

**Authors:** Adewale O Obadina, Olusola B Oyewole, Oluwasolabomi E Williams

**Affiliations:** Department of Food Science and Technology, Federal University of AgricultureAbeokuta, Nigeria

**Keywords:** Ajogun, cassava, extruded snack, nutrition

## Abstract

This study was carried out to investigate and improve the traditional processing method and nutritional quality of the traditional cassava snack (Ajogun). Cassava root (*Manihot esculenta* Crantz L.) of TME 419 variety was processed into mash (40% moisture content). The cassava mash was mixed into different blends to produce fried traditional “Ajogun”, fried and baked extrudates (modified Ajogun) as snacks. These products were analyzed to determine the proximate composition including carbohydrate, fat, protein, fiber, ash, and moisture contents and functional properties such as bulk density. The results obtained for the moisture, fat, protein, and ash contents showed significant difference (*P* < 0.05) between the control sample and the extrudates. However, there was no significant difference (*P* > 0.05) in the carbohydrate and fiber contents between the three samples. There was no significant difference (*P* > 0.05) in the bulk density of the snacks. Also, sensory evaluation was carried out on the cassava-based snacks using the 9-point hedonic scale to determine the degree of acceptability. Results obtained showed significant difference (*P* < 0.05) between the extrudates and control sample in terms of appearance, taste, flavor, color, aroma, texture, and overall acceptability. The highest acceptability level of the product was at 8.04 for the control sample (traditional Ajogun). This study has shown that “Ajogun”, which is a lesser known cassava product, is rich in protein and fat.

## Introduction

Food extrusion is a process in which a food material is forced to flow under one or more varieties of conditions of mixing, heating, and shearing through a die that is designed to form and/or puff-dry the ingredients (Rossen and Miller 1973). It is a very important high-temperature short-time process in producing fiber-rich products, and in producing a wide range of products such as breakfast cereals, snacks, pet foods, and texturized vegetable protein from starchy food material. Extrusion technology provides the opportunity to process a variety of snack products by just changing a minor ingredient and processing condition on the same machine. Several different shapes, textures, and colors of snack foods are possible by using an extruder. The extrusion process can produce innovative snacks which capture the consumer's imagination. While extrusion processing is not new, many novel foods are now produced using this technology and it is being increasingly applied to enable conventional foods to be produced either with improved functional properties or with significant cost savings. In its simplest form, the pasta is rolled out into a sheet and cut into strips, which are either cooked immediately or allowed to dry and kept for later consumption.

Pelembe et al. (2002) reported that, in Africa, due to deforestation by utilization of wood for fuel, there is a great need for precooked foods. According to Brennan (2006), there are many benefits to using extruders to process food materials. Extrusion systems are able to process highly viscous materials that are difficult or impossible to handle using conventional methods. The ability of extrusion systems to carry out a series of unit operations simultaneously and continuously gives rise to savings in labor costs, floor space costs, and energy costs while increasing productivity. Besides processing advantages, extrusion cooking can also induce some beneficial nutritional and chemical changes in foods (Camire 2002). Research has been carried out on the extrusion cooking of cereals and legumes (Pelembe et al. 2002; Ding et al. 2006) but research on high temperature short time extrusion cooking of cassava mash is scarce. The product's color, flavor, shape, and texture are also affected by the extrusion process.

Cassava (*Manihot esculenta* Crantz L) is an important food crop in the tropics and is a major carbohydrate staple. According to the Food and Agriculture Organization, cassava is the third most important source of calories in the tropics, after rice and corn (Food and Agriculture Organization 2002). It is usually processed traditionally to obtain different relatively shelf stable intermediate and final products for various food applications. These products include “gari,” a roasted fermented cassava meal, “agbelima,” which is a fermented cassava mash, and the dried cassava chips known as “kokonte,” which is further processed into cassava flour. Tapioca is a minor product or by-product from cassava processing. For industrial use, cassava is processed to obtain starch. This intermediate product can be used for producing sugar, acetone, and alcohol. Some people believe that Brazil can produce 20% of the alcohol it needs for motor fuel from cassava.

“Ajogun” is a traditional cassava-based fried snack product which is popular in Badagry area of Lagos State, Nigeria. It is a little known traditional product of cassava on which little or no investigation has been carried out. The need to improve the technology of cassava processing has been identified (Oyewole 2003). Some of the ways of standardizing or modifying the traditional process of producing “Ajogun” (traditional fried cassava snack) is introduction of extrusion technology and ingredients that will nutritionally add value to the traditional snack. The objectives of this research work were to investigate and improve the traditional processing method, analyze the nutritional quality of the traditional cassava snack (Ajogun), and the consumer's acceptability of the improved product.

## Materials and Methods

### Preliminary survey

In order to study the traditional processing technology of “Ajogun” production from cassava mash, two survey visits were made to Badagry in Lagos State, Nigeria, where the product is majorly consumed. Vocal group discussion was employed to obtain information on how the traditional processing is carried out and the production process employed by the processors was observed it is applicable only to African countries.

### Source of materials

Cassava root (*M. esculenta*) of variety TME 419 of cyanogenic potential (CNP) of 6.33 ppm was freshly harvested from a farm in Isolu, Abeokuta. It was processed into Cassava mash within 48 h of harvesting. The dry ingredients such as powdered spices (turmeric, sucrose, salt, chili, and monosodium glutamate), liquid onion, egg white, vegetable fat, and vegetable oil were purchased from a local market within Abeokuta, Ogun State, Nigeria.

### Methods

#### Production of cassava mash

The cassava roots of TME 419 variety were washed, peeled, and rewashed with clean water. The roots were immediately grated into pulp, which was dewatered with the aid of a hydraulic press for 40–60 min. The semidried pulp was then sieved with clean woven sieve to remove fibrous materials. The sieved pulp was packed in a clean container for the snack production to commence.

### Traditional processing of cassava mash to “Ajogun”

**Figure 1:**
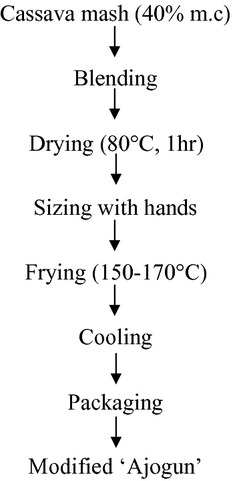
Flowchart for the production of traditional Ajogun.

The cassava mash was weighed and blended with weighed ingredients (water, sugar, salt, and liquid onion). The mix was left to equilibrate at 25°C after which it was taken in a small portion per time, molded with the palm of the hand into the desired shape, and fried in hot vegetable oil at 150–170°C. The fried cassava-based snack was cooled and packaged in an air-tight polyethylene bag. The formula preparation of the traditional “Ajogun” is shown in Table 1 and the production process is outlined in Figure 1.

**Table 1:** The formula preparation of traditional “Ajogun”

Ingredients	Weight (g)
Cassava mash	100
Sugar	50
Salt	50
Liquid onion	60
Fat	20
Egg	30

### Modified processing methods of cassava mash to “Ajogun”

**Figure 2:**
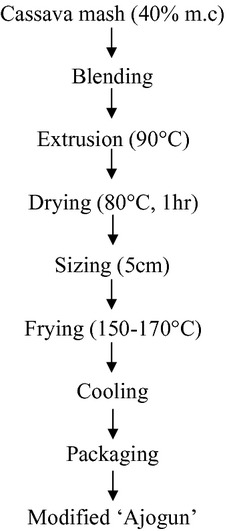
Flowchart for the production of fried cassava-based extrudate.

**Figure 3:**
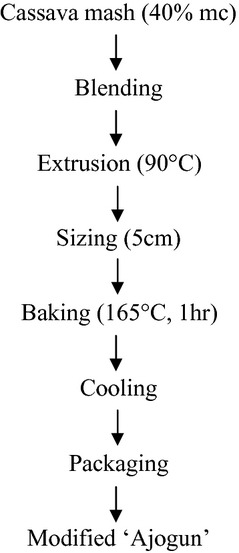
Flowchart for the production of baked cassava-based extrudate.

All the ingredients used were weighed in the appropriate proportion needed. The cassava mash was weighed in two cleaned bowls and the weighed ingredients were mixed and blended with the mash after which the feed sample of each formula was left to equilibrate (to be evenly balanced) at 25°C for 30 min before extrusion. After precooking in the extruder at 90°C, the first extrudates were collected, dried at 80°C for 1 h in a cabinet drier, cut into 5 cm pieces, fried for 10 min in hot oil under atmospheric pressure, cooled down, and packaged. The second extrudates were collected, cut into 5 cm pieces, baked in a hot-air oven at 165°C for 1 h, cooled down, and packaged. The formula preparation of the extrudates is shown in Table 1 and the production of these two extrudate samples is outlined in the flowcharts (Figs. 2 and 3 respectively).

### Analysis of cassava-based snacks

#### Determination of moisture content

The method described by AOAC (1990) was adopted.


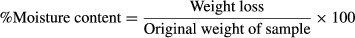


#### Determination of ash content

The AOAC (1990) method was used.


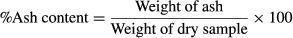


#### Determination of crude fiber

The method described by AOAC (1990) was used.


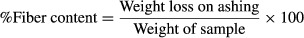


#### Determination of crude protein

The micro-Kjeldahl method as described by AOAC (1990) was used.


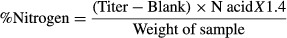



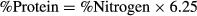


#### Determination of carbohydrate

The total carbohydrate content was determined by difference method, that is, the sum of the percentage moisture, ash, crude lipid, crude protein and crude fiber was subtracted from 100%.


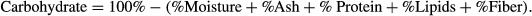


#### Bulk density determination

The ground sample (8% moisture content) was placed in a 100 mL measuring cylinder. The cylinder was tapped continuously until a constant volume was obtained. The bulk density was calculated as the weight of the ground sample (g) divided by grit volume (mL) (Okaka and Potter 1979).

#### Sensory evaluation

The cassava extrudates and the control sample (traditionally prepared “Ajogun”) were prepared and served to 24 taste panelists, all students of the University community. A descriptive 9-point hedonic scale rating was used to score samples for color, taste, texture, flavor, aroma, appearance, and general acceptability with 9 as like extremely, 1 as dislike extremely, and 5 neither like nor dislike.

### Statistical analysis

The results obtained were analyzed statistically using analyses of variance (ANOVA) to know its significance. One-way ANOVA and least significant difference were used to compare means.

## Results and Discussion

### Proximate composition of the cassava-based snacks

#### Carbohydrate content

The carbohydrate contents of the control sample and the cassava-based extrudates are shown in Table 2. The carbohydrate content of these extrudates and the control sample ranges from 35.35 ± 3.81 to 51.14 ± 5.44. There is no significant difference (*P* > 0.05) in the carbohydrate content of these extrudates and the control sample. The sample EA2 has the highest carbohydrate content while sample EA1 has the least carbohydrate content. This was due to the fact that the samples were composed of mainly carbohydrate rich materials, which are cassava mash and sugar.

**Table 2:** Proximate composition of cassava-based snacks

	Sample codes		
	
	TA1	EA1	EA2
Moisture (%)	2.90^a^ ± 0.14	7.70^b^ ± 0.42	8.60^c^ ± 0.00
Fat (%)	24.68^b^ ± 2.44	25.80^b^ ± 0.64	20.60^a^ ± 1.06
Protein (%)	8.28^a^ ± 0.16	9.01^ab^ ± 0.012	9.22^b^ ± 0.45
Ash (%)	2.50^ab^ ± 0.21	2.00^a^ ± 0.35	2.90^b^ ± 0.00
Fiber (%)	3.20^a^ ± 2.69	3.20^a^ ± 2.40	3.20.55^a^ ± 4.83
Carbohydrate (%)	40.91^a^ ± 5.64	35.35^a^ ± 3.81	51.14^a^ ± 5.44

All values are means of triplicate determinations ± standard deviation (SD). Means with different superscripts within the same rows are significantly different (*P* < 0.05). TA1, traditional “Ajogun” (fried); EA1, extruded “Ajogun” (fried); EA2, extruded “Ajogun” (baked).

#### Crude protein content

The protein contents of the control sample and the extrudates are shown in Table 2. The percentage of protein contents of the control sample and extrudates ranges from 8.28 ± 0.16 to 9.22 ± 0.455. There is a significant difference (*P* < 0.05) between the protein content of the three samples. The sample TA1 has the least percentage of protein content while sample EA2 has the highest percentage of protein content. This is as a result of the addition of egg white to the extrudates.

#### Fat content

The crude fat content of the control sample and extrudate products is shown in Table 2. The fat content ranges from 25.80 ± 0.64 to 14.60 ± 1.06. The result shows that sample EA1 has the highest percentage of fat content while sample EA2 has the least percentage of fat content. The fat content of sample EA2 is significantly different (*P* < 0.05) from TA1 and EA1. However, there is no significant difference (*P* > 0.05) between sample TA1 and EA1. This is the result of the deep fat frying process that both TA1 and EA1 went through. Fat plays a significant role in the shelf life of food products and as such a relatively high fat content could be undesirable in fried and baked food products. This is because fat can promote rancidity in foods, leading to the development of unpleasant and odorous compounds (Ihekoronye and Ngoddy 1985).

#### Ash content

The ash content of the control sample and the extrudate products is shown in Table 2. The ash content ranges from 2.00 ± 0.35 to 2.90 ± 0.00. The sample EA1 is significantly different (*P* < 0.05) from sample EA2 while sample TA1 is not significantly different (*P* > 0.05) from EA1 and EA2. This result shows that sample EA2 has the highest percentage of ash content while sample EA1 has the least percentage of ash content. This is as a result of the baking process that sample EA2 went through. It then follows that incorporation of baking process in the production of cassava-based snacks could enhance the mineral intake of many people, as ash is indicative of the amount of minerals contained in any food sample.

#### Fiber content

The fiber content of the control sample and the extrudates is shown in Table 2. The fiber content ranges from 13.55 ± 4.83 to 20.73 ± 2.69. The result shows that there is no significant difference (*P* > 0.05) in the fiber content of the three samples. This indicates that both the control sample and the extrudates are high in percentage of crude fiber. This is due to the high fiber content of cassava mash. Crude fiber is known to aid the digestive system of humans (Ihekoronye and Ngoddy 1985), indicating that “Ajogun”, a cassava-based snack, could find acceptability by many people as well as by health organizations.

#### Moisture content

The moisture content of the control sample and extrudates is shown in Table 2. The moisture content ranges from 2.90 ± 0.14 to 8.60 ± 0.00. There is significant difference (*P* < 0.05) between the moisture content of the three samples. Sample TA1 has the least percentage of moisture content while sample EA2 has the highest value. This is due to sample TA1's shape and large surface area. These values were minimal and may not have an adverse effect on the quality attributes of the product (Kure et al. 1998).

#### Bulk density

The bulk density of the cassava-based samples ranged from 0.71 ± 0.06 to 0.61 ± 0.0087 (Table 3). There is no significant difference (*P* > 0.05) in the bulk density of the three samples. Bulk density gives an indication of the relative volume of packaging material required and high bulk density is a good physical attribute when determining the mixing quality of a particulate matter (Achinewhu et al. 1998). The bulk density is a reflection of the load the samples can carry, if allowed to rest directly on one another. The density of processed products dictates the characteristics of its container or package. The product's density influences the amount and strength of packaging material, texture, or mouth feel (Lewis 1990). Stojceska et al. (2009) reported that bulk density is highly correlated with the moisture content of the product during extrusion.

**Table 3:** Extrudate characteristics

Sample code	Bulk density (g/mL)
TA1	0.71^a^ ± 0.06
EA1	0.61^a^ ± 0.02
EA2	0.61^a^ ± 0.009

All values are means of triplicate determinations ± standard deviation (SD). Means with the same superscripts within the same column are not significantly different (*P* > 0.05). TA1, traditional “Ajogun” (fried); EA1, extruded “Ajogun” (fried); EA2, extruded “Ajogun” (baked).

### Sensory evaluation of the cassava-based snacks

#### Color

Color is an important sensory attribute of any food because of its influence on acceptability. Color is an important parameter in judging properly fried and baked snacks as golden to brown color resulting from Maillard reaction is always associated with fried and baked products. It also shows the suitability of the raw material used for the preparation, provides information about the formation and quality of the product. Mean quality score of the color of the snacks is given in Table 4. The samples scored between 7.63 and 5.83 on the 9-point hedonic scale, indicating that the color of the snacks was liked moderately and agree with the observations of Iwe (2007). The color of sample TA1 is significantly different (*P* < 0.05) from the color of the extrudates. It is evident from the result that TA1, which was locally produced, significantly (*P* < 0.05) has the highest mean score while EA1, the fried extrudate, significantly (*P* < 0.05) has the lowest mean score. Judges shows a slight preference sample EA1, which is the fried extrudate.

**Table 4:** Sensory attributes of cassava-based snacks

Sample code	TA1	EA1	EA2
Color	7.63^b^ ± 1.01	5.83^a^ ± 1.69	6.25^a^ ± 1.92
Flavor	7.50^b^ ± 1.22	6.42^a^ ± 1.38	6.29^a^ ± 1.55
Taste	8.04^b^ ± 0.79	6.00^a^ ± 1.84	5.88^a^ ± 1.77
Appearance	8.13^b^ ± 0.99	5.54^a^ ± 1.88	6.38^a^ ± 1.83
Aroma	7.54^b^ ± 1.14	6.17^a^ ± 1.63	6.08^a^ ± 1.53
Texture	7.83^b^ ± 1.31	5.50^a^ ± 1.53	5.96^a^ ± 1.94
Overall acceptability	8.04^b^ ± 0.95	5.88^a^ ± 1.83	6.08^a^ ± 1.77

All values are means of triplicate determinations ± standard deviation (SD). Means with the same superscripts within the column are not significantly different (*P* > 0.05). TA1, traditional “Ajogun” (fried); EA1, extruded “Ajogun” (fried); EA2, extruded “Ajogun” (baked).

#### Flavor

Flavor is the main criterion that makes the product to be liked or disliked. Quality score for the flavor of the snacks revealed that the flavor varied significantly (*P* < 0.05) between the control sample and the extrudates. The results in Table 4 indicate that sample TA1 prepared traditionally from cassava mash had a maximum score (7.5) for flavor. With respect to the flavor, the judges accepted snacks prepared from cassava mash subjected to both traditional techniques and technological processes.

#### Appearance

Appearance is a desirable quality of snacks. Table 4 shows the quality scores for the appearance of snacks, the results of which ranged from 8.13 to 5.54. The control sample (TA1), with the highest score of 8.13, is significantly different (*P* < 0.05) from the extrudates. Judges liked the appearances of EA1 and EA2 snacks. The extrusion process was obviously responsible for the lower scores and poor appearances. The effect of processes such as extrusion, frying, and baking on the appearance of snacks is well known.

#### Texture

The texture of the snacks containing cassava mash is shown in Table 4. The textures of the samples were significantly affected due to the high percentage of fiber content of cassava. Sample TA1 prepared from the traditional process had the highest score of 8.7 which is significantly different (*P* < 0.05) from samples EA1 and EA2 with low mean scores of texture which were produced by a technological process. With respect to the texture, judges accepted snack TA1. The effects of fat and fiber contents on the texture of snack are well known.

#### Taste

The taste of the snacks containing cassava mash in their formulation is shown in Table 4. The results show that the ingredients, particularly the sweeteners and powdered spices, significantly (*P* < 0.05) affected the taste of the snacks. Sample TA1 had the maximum score of 8.04 while the extrudates had the minimum score of 6.0 and 5.87 on the 9-point hedonic scale. These indicate that the taste of sample TA1 was liked very much while the tastes of samples EA1 and EA2 were slightly liked.

#### Overall acceptability

The statistical analysis regarding the overall acceptability of snacks prepared from cassava mash is shown in Table 4. The results show that the ingredients used significantly (*P* < 0.05) affected the overall acceptability of the snacks. The control sample (TA1) had the maximum score (8.04) while minimum scores (6.08) and (5.87) were scored by the EA2 and EA1 snacks (Table 4). Snacks prepared from extrusion process were slightly accepted by judges with respect to overall acceptability.

## Conclusion

The result of the study has shown that “Ajogun”, which is a lesser known cassava product, is rich in protein and fat. The result of the sensory analyses showed that the appearance, texture, and taste indeed influenced the overall acceptability of this cassava-based product by the panel of consumers. The traditional processing shows that new nutritional additives could be incorporated into the recipe. This study has opened up new possibilities for improving the traditional technique of producing “Ajogun”.

## References

[b1] Achinewhu SC, Barber LI, Ijeoma IO (1998). Physico-chemical properties and garrification of selected cassava cultivars in River State. Plant Food Hum. Nutr.

[b501] AOAC (1990). Official methods of analysis.

[b3] Brennan JG (2006). Food processing handbook EDICION. Wiley-VCH.

[b4] Camire ME, Chaovanalikit A, Dougherty MP, Briggs J (2002). Blueberry and grape anthocyanins as breakfast cereal colorants. J. Food Sci.

[b6] Ding EL, Hutflessi SM, Ding X, Girotra S (2006). Chocolate and prevention of cardiovascular disease: a systematic review. Nutr. Metab.

[b7] Food and Agriculture Organization (2002). The cassava transformation in Africa.

[b8] Ihekoronye AT, Ngoddy PO (1985). Integrated food science and technology for the tropics.

[b9] Iwe MO (2007). Current trends in sensory evaluation of foods.

[b502] Kure OA, Bahago EJ, Daniel EA (1998). Studies on the proximate composition and effect of flour particle size on acceptability of biscuit produced from blends of soyabeans and plantain flours. Namida Tech-Scope J.

[b10] Lewis MJ (1990). Physical properties of food and its processing systems.

[b503] Okaka JC, Potter NW (1979). Physicochemical and functional properties of cowpea powders processed to reduce beany flavours. J. Food Sc.

[b11] Oyewole OB (2003). Development of the small and medium scale enterprises sector producing cassava-base products. http://www.thisdayonline.com/archive/2003/19edu02html.

[b12] Pelembe LA, Erasmus C, Taylor JR (2002). Development of a protein-rich composite sorghum-cowpea instant porridge by extrusion cooking process. Lebenson Wiss U Technol.

[b14] Rossen JL, Miller RC (1973). Food extrusion. Food Technol.

[b15] Stojceska V, Ainsworth P, Plunkett A, Ïbanoglu S (2009). The effect of extrusion cooking using different water feed rates on the quality of ready-to-eat snacks made from food by-products. Food Chem. J.

